# In Vitro Synergistic Inhibition of HT-29 Proliferation and 2H-11 and HUVEC Tubulogenesis by Bacopaside I and II Is Associated with Ca^2+^ Flux and Loss of Plasma Membrane Integrity

**DOI:** 10.3390/ph14050436

**Published:** 2021-05-06

**Authors:** Yoko Tomita, Eric Smith, Helen M. Palethorpe, Maryam Nakhjavani, Kenny K. L. Yeo, Amanda R. Townsend, Timothy J. Price, Andrea J. Yool, Jennifer E. Hardingham

**Affiliations:** 1 Solid Tumour Group, Basil Hetzel Institute for Translational Health Research, The Queen Elizabeth Hospital, Woodville South, SA 5011, Australia; eric.smith@adelaide.edu.au (E.S.); helen.palethorpe@unisa.edu.au (H.M.P.); maryam.nakhjavani@adelaide.edu.au (M.N.); kenny.yeo@student.adelaide.edu.au (K.K.L.Y.); amanda.townsend@sa.gov.au (A.R.T.); timothy.price@sa.gov.au (T.J.P.); jennifer.hardingham@adelaide.edu.au (J.E.H.); 2 Adelaide Medical School, University of Adelaide, Adelaide, SA 5005, Australia; andrea.yool@adelaide.edu.au; 3 Department of Medical Oncology, The Queen Elizabeth Hospital, Woodville South, SA 5011, Australia

**Keywords:** bacopaside I, bacopaside II, colorectal cancer cells, endothelial cells, proliferation, migration, tube formation, apoptosis, Ca^2+^ flux, plasma membrane

## Abstract

We previously showed how triterpene saponin bacopaside (bac) II, purified from the medicinal herb *Bacopa monnieri*, induced cell death in colorectal cancer cell lines and reduced endothelial cell migration and tube formation, and further demonstrated a synergistic effect of a combination of bac I and bac II on the inhibition of breast cancer cell line growth. Here, we assessed the effects of bac I and II on the colorectal cancer HT-29 cell line, and mouse (2H-11) and human umbilical vein endothelial cell (HUVEC) lines, measuring outcomes including cell viability, proliferation, migration, tube formation, apoptosis, cytosolic Ca^2+^ levels and plasma membrane integrity. Combined bac I and II, each applied at concentrations below IC_50_ values, caused a synergistic reduction of the viability and proliferation of HT-29 and endothelial cells, and impaired the migration of HT-29 and tube formation of endothelial cells. A significant enhancement of apoptosis was induced only in HUVEC, although an increase in cytosolic Ca^2+^ was detected in all three cell lines. Plasma membrane integrity was compromised in 2H-11 and HUVEC, as determined by an increase in propidium iodide staining, which was preceded by Ca^2+^ flux. These in vitro findings support further research into the mechanisms of action of the combined compounds for potential clinical use.

## 1. Introduction

Herbal medicine has been used for thousands of years in many societies and takes advantage of the inherent healing properties of plants. It is still a mainstay of primary health care in many parts of the world, and is also used to complement more conventional medicine [1]. Many herbal products have been shown to demonstrate cytotoxicity using in vitro and in vivo models of cancer, and some chemotherapy drugs in clinical use were developed from plant extracts [2]. Vincristine, derived from the periwinkle plant, and taxanes, derived from the bark of the Pacific yew tree, are some examples [3].

Bacopaside (bac) I and bac II are methanol extracts of *Bacopa monnieri* (BM), a herbal plant used in Ayurvedic medicine as a cognitive enhancer, sedative and anti-epileptic [4]. They belong to plant terpenoids, triterpenoid saponins, which have attracted interest as novel natural anticancer compounds with favorable efficacy and safety profiles. Several triterpenoid saponins, including Jujuboside B, senegin III and SB365, have been shown to exhibit anticancer activity using in vivo models [5,6,7].

The current literature supports the anticancer effect of BM extracts; however, the exact extracts evaluated vary between studies. Ghosh et al. reported treatment with stigmasterol, a phytosterol isolated from aerial parts of BM, reduced tumour volume and increased survival of mice injected intraperitoneally with Ehrlich Ascites Carcinoma (EAC) [8]. Mallick et al. examined the antiproliferative effect of ethanol extracts of BM on cancer cell lines of multiple origins, and demonstrated that a dichloromethane (DCM) fraction of the extract exhibited the maximal cytotoxic activity among all fractions [9,10]. Oral administration of a DCM fraction was further shown to reduce tumour volume compared to no treatment in mice inoculated with EAC intraperitoneally [10].

Our group showed previously that bac II inhibited the proliferation of colon cancer cells through the induction of cell cycle arrest and cell death, and bac I and II synergistically impaired proliferation, migration and invasion of breast cancer cells [11,12]. We additionally showed that migration and tube-formation of endothelial cells were inhibited by bac II, and proposed it has potential anti-angiogenic efficacy [13]. The current study aimed to assess the anticancer properties of the combination of bac I and II on colon cancer and endothelial cells in vitro, with a hypothesis that they exhibit synergistic cytotoxicity. This is the first time the anti-angiogenic property of bac I, with or without bac II, has been reported.

Bac I and II are proven modulators of aquaporin 1 (AQP1), a transmembrane protein with water and ion channel functions, which has been implicated in tumour proliferation, migration, and angiogenesis [14,15]. Our group has established a murine subcutaneous tumour model using moderately AQP1-expressing HT-29 colon cancer cells for evaluation of AQP1 modulators. The study was performed as groundwork for in vivo testing of combined bac I and II in this murine model and evaluated the antiproliferative and antimigratory effect of this combined treatment on HT-29 colon cancer cells, as well as its anti-tubulogenic effect on 2H-11 mouse endothelial cells and human umbilical endothelial cells (HUVEC). The murine endothelial cell line was specifically chosen to simulate the animal model in cell culture. The mechanism underlying the anticancer property of the combination of bac I and II was further explored by focusing on apoptosis induction, cytosolic Ca^2+^ and plasma membrane integrity.

## 2. Results

### 2.1. IC_50_ Values for Bac I and II, Singly and Combined, Differed between Cell Lines

**Figure 1:**
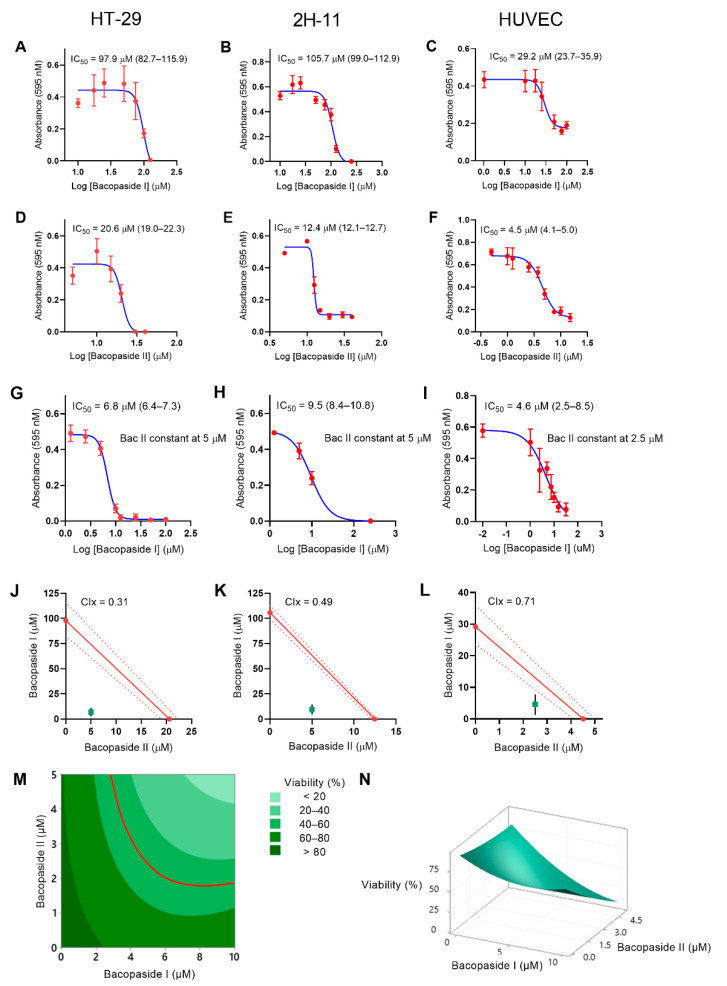
Comparison of the half maximal inhibitory concentration (IC_50_) values of bac I and II, alone and combined, and their combination index (CIx) in HT-29, 2H-11 and HUVEC. Cell growth was measured by a crystal violet assay and IC_50_ was determined by nonlinear regression analysis of 6 replicates. Dose–response curves show IC_50_ for bac I (**A**–**C**), bac II (**D**–**F**) and combinations (**G**–**I**). Error bars represent the 95% confidence interval (CI). On the isobolograms (**J**–**L**), theoretical IC_50_ lines (red solid lines) acquired from the IC_50_ for each compound used individually, and the measured IC_50_ (green square) for bac I and II combined, are plotted for each cell line, with 95% CI being represented by the red dotted lines for theoretical IC_50_ lines and by the black lines for measured IC_50_. Response surface methodology (RSM) analysis was performed on HUVEC viability, and the contour plot (**M**) and surface plot (**N**) are drawn, with the red line indicating the 50% probability isobole.

A crystal violet growth assay was performed on HT-29, 2H-11 and HUVEC following treatment with bac I and II, either alone or in combination. There was a dose–response effect with both compounds (Figure 1 and Table 1). For HT-29, the half maximal inhibitory concentration (IC_50_) was 97.9 μM (95% CI 82.7–115.9 μM) for bac I (Figure 1A), and 20.6 μM (95% CI 19.0–22.3 μM) for bac II (Figure 1D). Bac II exhibited more cytotoxicity on 2H-11 than HT-29, and the IC_50_ was 105.7 μM (95% CI 99.0–115.9 μM) for bac I (Figure 1B) and 12.4 μM (95% CI 12.1–12.7) for bac II (Figure 1E). HUVEC was the most sensitive to the cytotoxicity of both bac I and II, and its IC_50_ was 29.2 μM (95% CI 23.7–35.9 μM) for bac I (Figure 1C) and 4.5 μM (95% CI 4.1–5.0 μM) for bac II (Figure 1F).

The IC_50_ of bac I and II combined was determined next. Bac II was the more potent compound, and was therefore kept constant at a concentration that did not reduce cell viability, whilst bac I concentration was altered. The concentration of bac II chosen was 5 μM for HT-29 and 2H-11, and 2.5 μM for HUVEC. Compared to the IC_50_ of bac I alone, the presence of bac II reduced the IC_50_ of bac I by more than 90% for HT-29 and 2H-11; from 97.9 μM to 6.8 μM (95% CI 76.4–7.3 μM) for HT-29 (Figure 1G) and from 105.7 μM to 9.5 μM (95% CI 8.4–10.8 μM) for 2H-11 (Figure 1H). For HUVEC, the presence of bac II reduced the IC_50_ of bac I by 84.2%, from 29.2 μM to 4.6 μM (95% CI 2.5–8.5 μM) (Figure 1I). These results were suggestive of compound synergism, and this was confirmed by the isobolograms, which showed the data points for the combination being plotted below the theoretical lines (Figure 1J–L). Combination indices (CIx) were less than one for all the cell lines: 0.31 for HT-29, 0.49 for 2H-11 and 0.71 for HUVEC. An additional response surface methodology (RSM) analysis performed on HUVEC viability showed a skewing of the 50% probability isobole towards the origin on the contour plot (Figure 1M), which is consistent with the synergistic interaction between bac I and II (Tables S1 and S2).

Based on these findings, concentrations of combined bac I and II, inclusive of those at and/or below the IC_50_s of combined treatment, were used to test their efficacy in subsequent functional assays. The highest and lowest concentrations of combined bac I and II (here on referred to as bac I/II to describe specific concentrations) chosen were bac I/II 10/5 µM (combined treatment IC_50_ for 2H-11) and 5/2.5 µM (combined treatment IC_50_ for HUVEC). We additionally examined bac I/II 5/5 and 10/2.5 µM and, for the HUVEC proliferation alone, bac I/II 2.5/2.5 μM was included. Bac I and II monotherapies at 10 and 5 μM, respectively, were examined in each assay for comparison, and again for the HUVEC proliferation assay alone bac II monotherapy at 2.5 μM was included.

### 2.2. Bac I and II Combined Impaired Cell Proliferation of Colon Cancer and Endothelial Cells

**Figure 2:**
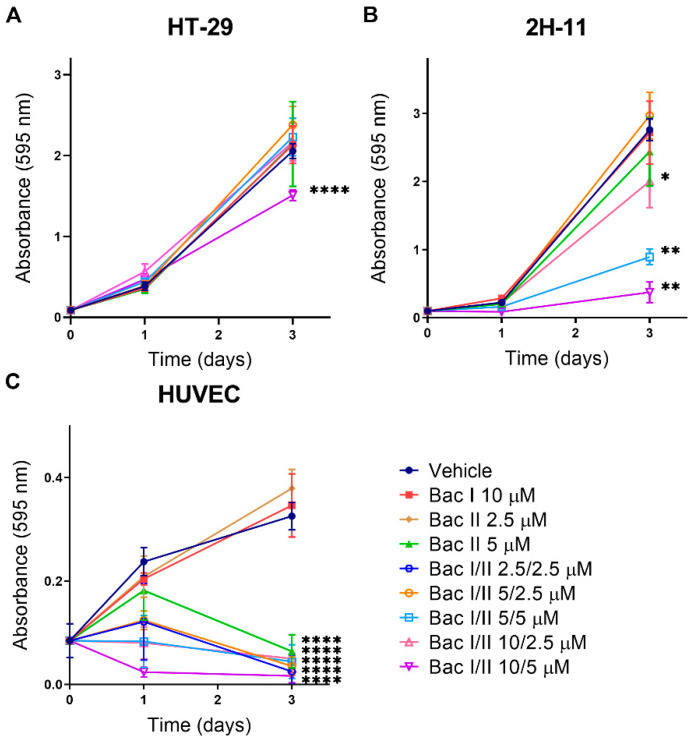
Bac I and II combined impaired proliferation of HT-29, 2H-11 and HUVEC. HT-29 (**A**), 2H-11 (**B**), and HUVEC (**C**) were treated with bac I and II, either alone or in combination, and cellular proliferation was measured over 3 days by a crystal violet assay. Data represent the mean absorbance of 5 to 6 replicates. Error bars represent the standard deviation (SD). Significant difference as compared to vehicle after 3-day treatment is indicated by asterisks (* *p* < 0.05; ** *p* < 0.01; **** *p* < 0.0001).

The cytotoxic effect of bac I and II, either alone or in combination, was examined in HT-29, 2H-11 and HUVEC by performing crystal violet proliferation assays (Figure 2). HT-29 was the most resistant; after 3 days of treatment, only bac I/II 10/5 μM inhibited proliferation by 26.5% (*p* < 0.0001), as compared to the vehicle (Figure 2A). In 2H-11, reduced proliferation was measured for bac I/II 5/5, 10/2.5, and 10/5 μM, resulting in 67.6% (*p* < 0.0001), 27.3% (*p* = 0.015), and 86.4% (*p* < 0.0001) inhibition compared to the vehicle, respectively (Figure 2B). Reduced proliferation was measured for HUVEC treated with all the combination concentrations, but not with bac I and II monotherapies by day 1; bac I/II 2.5/2.5 μM, the lowest combination concentration tested, inhibited proliferation by 48.8% compared to the vehicle (*p* < 0.0001) (Figure 2C). None of the combination concentrations tested showed an increase in the absorbance values on day 3 compared to day 0, implying a complete inhibition of HUVEC proliferation. On day 3, the inhibition of proliferation compared to the vehicle was additionally detected for bac II monotherapy at 5 μM (*p* < 0.0001), but not at 2.5 μM. The result was consistent with the IC_50_ of HUVEC, estimated to be 4.5 μM.

### 2.3. Bac I and II Combined Reduced Migration of Colon Cancer Cells

**Figure 3:**
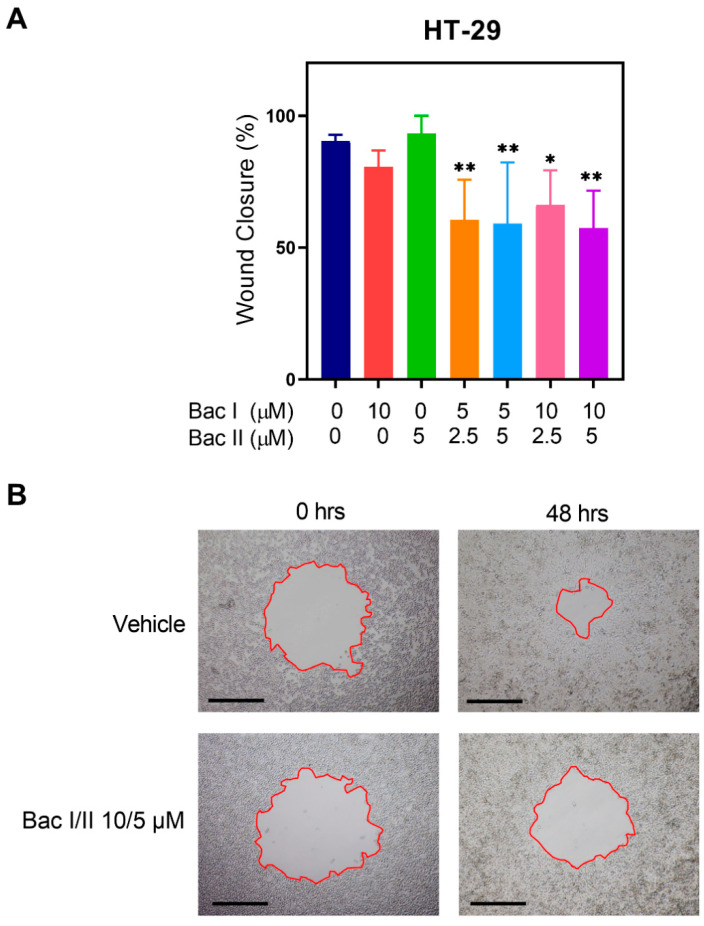
Bac I and II combined impaired migration of HT-29. (**A**) HT-29 was treated with bac I and bac II, either alone or in combination, and wound closure was measured after 48 h. Data represent the mean wound closure as a percentage of 5 to 6 replicates. Error bars represent the SD. Significant difference as compared to vehicle is indicated by asterisks (* *p* < 0.05; ** *p* < 0.01). (**B**) Representative images of scratch wounds at times 0 and 48 h are shown for treatment with vehicle and bac I/II 10/5 μM. Magnification ×40, scale bar = 0.5 mm.

The effect of bac I and II, either alone or in combination, on the migration of HT-29 was assessed using a circular scratch wound assay (Figure 3). After 48 h, treatment with bac I/II 5/2.5, 5/5, 10/2.5 and 10/5 μM resulted in a 33.0% (*p* = 0.0047), 34.7% (*p* = 0.0045), 26.7% (*p* = 0.0368) and 36.6% (*p* = 0.0026) reduction in wound closure compared to the vehicle, respectively. Migration was not significantly altered by either bac I 10 μM or bac II 5 μM.

### 2.4. Bac I and II Combined Reduced Endothelial Cell Tube Formation

**Figure 4:**
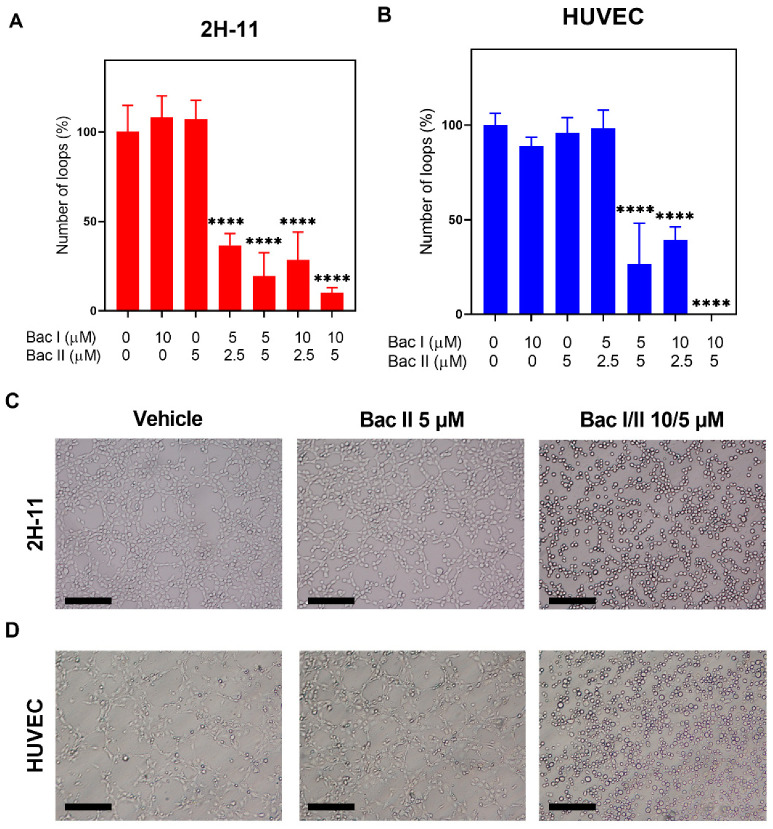
Bac I and II combined impaired tube formation of 2H-11 and HUVEC. 2H-11 (**A**) and HUVEC (**B**) were treated with bac I and bac II, either alone or in combination, and the number of loops formed was counted after 2 h. Data represent the mean percentage in the number of loops formed, with respect to the vehicle for triplicates. Error bars represent the SD. Significant difference as compared to vehicle is indicated by asterisks (**** *p* < 0.0001). Representative images of 2H-11 (**C**) and HUVEC (**D**) treated with the vehicle, bac II 5 µM and bac I/II 10/5 µM are shown. Magnification 100×, scale bar = 0.2 mm.

The effect of bac I and II, either alone or in combination, was tested on tube formation of 2H-11 and HUVEC (Figure 4). For 2H-11, the number of loops formed was reduced by 63.4%, 80.4%, 71.5% and 89.8% for bac I/II 5/2.5, 5/5, 10/2.5 and 10/5 μM, respectively, compared to the vehicle (*p* < 0.0001) (Figure 4A). No inhibition of tube formation was detected with either bac I 10 μM or bac II 5 μM. For HUVEC, the number of loops formed was reduced by 60.6%, 73.3% and 100% for bac I/II 5/5, 10/2.5 and 10/5 μM, respectively, compared to the vehicle (*p* < 0.0001) (Figure 4B). Treatment with bac I or II alone was not associated with the inhibition of tube formation.

### 2.5. Bac I and II Combined Induced Activation of Caspase 3 and 7 in HUVEC, but Not in HT-29

**Figure 5:**
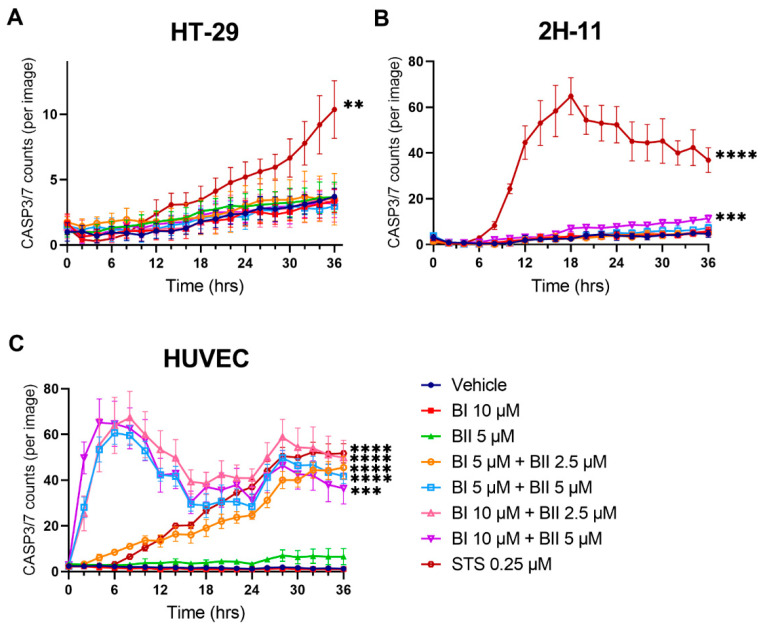
Bac I and II combined induced caspase 3/7 activation in HUVEC, but not in HT-29. HT-29 (**A**), 2H-11 (**B**) and HUVEC (**C**) were treated with bac I and II, either alone or in combination, and caspase 3/7 activation was monitored using CellEvent Caspase-3/7 Green Detection Reagent. Data represent the mean number of caspase 3/7-positive cells per image for 6 replicates. Error bars represent the SD. A significant difference at 36 h as compared to the vehicle is indicated by asterisks (** *p* < 0.01; *** *p* < 0.001 **** *p* < 0.0001).

Based on our previous finding that bac II caused induction of annexin V positivity in colon cancer and endothelial cells [11,13], it was hypothesised that the combination of bac I and II similarly triggers cell death. We conducted kinetic live-cell imaging assays using CellEvent Caspase-3/7 Green Detection Reagent to evaluate caspase 3/7 activation in response to treatment with bac I and/or II (Figure 5). For all the cell lines tested, the vehicle caused a minimal activation of caspase 3/7. In HT-29, unlike the positive control (staurosporine), bac I and II, neither alone nor in combination, resulted in a significant increase in caspase 3/7 activation compared to the vehicle, indicating a lack of induction of apoptosis (Figure 5A). A similar result was measured for 2H-11, except that bac I/II 10/5 μM caused a small rise in the number of caspase 3/7-positive cells compared to the vehicle. At 36 h, caspase 3/7 activation was 2.4 times higher for bac I/II 10/5 μM compared to the vehicle (*p* < 0.0001) (Figure 5B). HUVEC were the most sensitive cells (Figure 5C), and an increase in the number of caspase 3/7-positive cells compared to the vehicle was found as early as 2 h for bac I/II 5/5, 10/2.5 and 10/5 μM. At 36 h, caspase 3/7 activation was increased by a factor of 35.2 for bac I/II 5/2.5 μM (*p* < 0.0001), 32.3 for 5/5 μM (*p* < 0.0001), 38.6 for 10/2.5 μM (*p* < 0.0001) and 28.2 for 10/5 μM (*p* = 0.0002) compared to the vehicle.

### 2.6. Bac I and II Combined Induced Calcium Flux in Colon Cancer and Endothelial Cells

**Figure 6:**
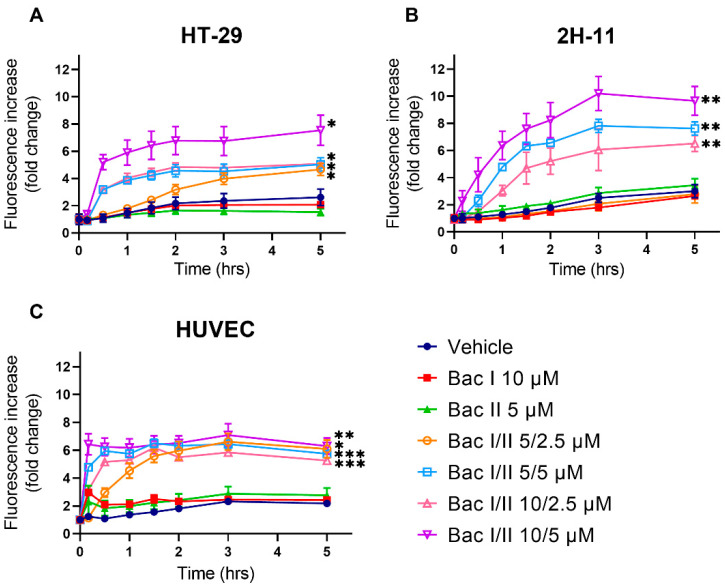
Bac I and II combined induced an increase in cytosolic Ca^2+^ in HT-29, 2H-11 and HUVEC. HT-29 (**A**), 2H-11 (**B**) and HUVEC (**C**) were treated with bac I and II, either alone or in combination, and cytosolic Ca^2+^ was monitored over 5 h by a Fluo-8 assay. Data represent the mean fractional increase in fluorescence with respect to time 0 of triplicates. Error bars represent the SD. Significant difference as compared with the vehicle after 5-h of treatment is indicated by asterisks * *p* < 0.05; ** *p* < 0.01; *** *p* < 0.001).

The influx of Ca^2+^ is a known trigger for the activation of both intrinsic and extrinsic apoptosis pathways [16]. We tested the effect of bac I and II, either alone or in combination, on cytosolic Ca^2+^ in HT-29, 2H-11 and HUVEC using a Fluo-8 Ca^2+^ flux assay (Figure 6). For HT-29, the amount of cytosolic Ca^2+^ at 5 h was higher for bac I/II 5/2.5, 5/5, 10/2.5 and 10/5 μM compared to the vehicle by a factor of 1.8 (*p* = 0.038), 1.9 (*p* = 0.023), 1.9 (*p* = 0.035) and 2.9 (*p* = 0.019), respectively (Figure 6A). Cytosolic Ca^2+^ similarly increased in 2H-11 treated with bac I and II combined. At 5 h, bac I/II 5/5, 10/2.5 and 10/5 μM resulted in 2.5-fold (*p* = 0.001), 2.2-fold (*p* = 0.006) and 3.2-fold (*p* = 0.009) higher cytosolic calcium compared to the vehicle, respectively (Figure 6B). For HUVEC, Ca^2+^ flux commenced more rapidly than that observed for HT-29 and 2H-11; for bac I/II 5/5, 10/2.5 and 10/5 μM, the majority of the increase in cytosolic Ca^2+^ was detected within 10 min of commencing treatment (Figure 6C). At 5 h, cytosolic Ca^2+^ measured for bac I/II 5/2.5, 5/5, 10/2.5 and 10/5 μM was higher than that of the vehicle by a factor of 2.8 (*p* = 0.016), 2.7 (*p* = 0.0002), 2.4 (*p* = 0.0005) and 2.9 (*p* = 0.01), respectively. Unlike HT-29 and 2H-11, HUVEC treated with bac I 10 μM and bac II 5 μM demonstrated an increase in calcium during the first 30 min.

### 2.7. Bac I and II Combined Was Associated with Disruption of the Plasma Membrane in Endothelial Cells, But Not in Colon Cancer Cells

**Figure 7:**
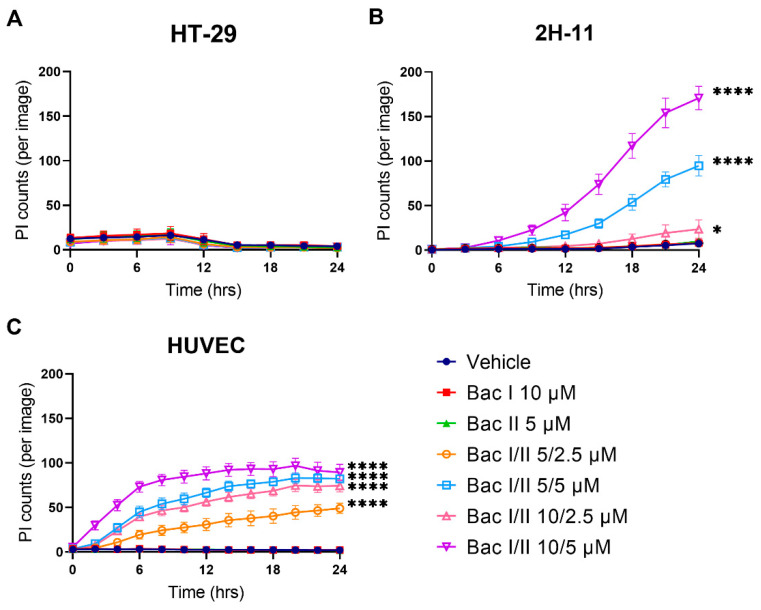
Disruption of the plasma membrane by bac I and II combined was cell line dependent. HT-29 (**A**), 2H-11 (**B**) and HUVEC (**C**) were treated with bac I and II, either alone or in combination, and the disruption of the plasma membrane was monitored over 24 h using propidium iodide (PI). Data represent the mean number of PI-stained cells per image for 6 replicates. Error bar represents SD. Significant difference as compared with vehicle after 24-h treatment is indicated by asterisks * *p* < 0.05; **** *p* < 0.0001).

Disruption of the cell membrane by combined bac I and II, as a cause of Ca^2+^ influx, was explored by serially monitoring treated cells and staining for propidium iodide (PI) (Figure 7). For HT-29, the number of PI-stained cells was very low, indicating minimal plasma membrane disruption caused in this cell line (Figure 7A). At 24 h, no statistically significant difference was detected in the number of PI-stained cells between the active treatment and the vehicle. For 2H-11, an increase in the number of PI-stained cells compared to the vehicle was seen for bac I/II 5/5, 10/2.5 and 10/5 μM, starting at 6, 12 and 3 h, respectively (Figure 6B). At 24 h, the number of PI-stained cells was higher than that for the vehicle by a factor of 13.0 (*p* < 0.0001), 3.1 (*p* = 0.046) and 31.7 (*p* < 0.0001) for 10/5 μM, for bac I/II 5/5, 10/2.5 and 10/5 μM, respectively. In contrast to HT-29 and 2H-11, plasma membrane disruption of HUVEC was induced much earlier for all combination concentrations (Figure 6C). At 24 h, the number of PI-stained cells was higher than that of the vehicle by a factor of 24.6, 41.1, 37.2 and 44.7, for bac I/II 5/2.5, 5/5, 10/2.5 and 10/5, respectively (*p* < 0.0001).

## 3. Discussion

Anticancer effects of BM extracts have been described elsewhere [17,18,19,20,21,22,23]. In vitro, ethanol and methanol extracts of BM exhibited cytotoxicity on mouse sarcoma cells, as well as human breast and prostate cancer cells [17,18,19]. Bac I decreased the viability of brain, breast, colon, lung, and prostate cancer cells, and impaired the adhesion, invasion and migration of breast cancer cells [20]. In vivo, the oral administration of bacoside A isolated from BM, which mainly contains bacoside A3, bac II, bacopaside X and bacopasaponin C, hindered the development and inhibited the growth of N-nitrosodiethylamine-induced hepatocellular carcinoma in albino rats [21]. Methanol extract of BM similarly delayed the development of dimethyl benz(a)anthracene plus croton oil-induced skin papilloma in swiss albino mice and reduced its incidence and the number of lesions [22,23]. An oral formulation of methanol extract of BM reduced the growth of subcutaneous B16F10 melanoma xenografts in C57BL mice [23].

In the current study, we not only showed the cytotoxicity of bac I and II, both as monotherapies and in combination, on HT-29 human colon cancer cells, but also on 2H-11 mouse endothelial cells and HUVEC, with tube formation of these two cell lines being inhibited by the combination. We previously reported that bac II 15 µM inhibited tube formation of 2H-11 and HUVEC [13]. This, however, is the first time the effect of bac I was examined on endothelial cells. The presence of bac I at 5 µM lowered the amount of bac II required to inhibit tube formation of 2H-11 and HUVEC to 2.5 and 5 µM, respectively, being consistent with our IC_50_ results. Impairment of tumour angiogenesis has proven clinical utility, and several anti-angiogenic compounds such as bevacizumab and apatinib are in clinical use, either in conjunction with chemotherapy agents or as a monotherapy. They are considered favourable over conventional chemotherapy agents in their target selectivity; hence, their better toxicity profile and limited physiological induction of angiogenesis minimises unwanted toxicities on vessels in a non-tumour environment [24]. It is additionally thought that they partially reverse structural and functional impairment associated with tumour vasculature and improve therapeutic response from chemotherapy agents when administered in combination [25]. The current finding strengthens the previously proposed therapeutic potential of combined bac I and II in cancer management [12].

Bac I and II combined were synergistic, inhibiting the proliferation of HT-29, 2H-11 and HUVEC in addition to the migration of HT-29 and tube formation of 2H-11, at concentrations below the IC_50_ of combined treatment. This is in line with our previous findings, where the viability of MDA-MB-231 triple negative breast cancer cells was impaired by bac I and II in a synergistic manner, and it was associated with inhibition of proliferation, migration and invasion at concentrations below the IC_50_ of combined treatment [12]. We and our collaborators previously reported bac I and II as monotherapies at 50 and 15 µM, respectively, inhibited the migration of HT-29, and bac II at 15 µM impaired tube formation of 2H-11 and HUVEC [13,14]. Therefore, the lack of functional inhibition observed with these monotherapies in the current study is at least partly explained by the low doses tested in the study. The synergism of the two compounds found here additionally suggests they target different proteins or signalling pathways to achieve functional inhibition of cancer and endothelial cells. Using cheminformatics and system pharmacological approaches, multitarget properties of various bioactive compounds of *Bacopa monnieri*, with frequent overlapping of the active targets, has been shown for neurological diseases [26].

In our previous work, lower concentrations of bac II relative to bac I were effective in inhibiting the cell viability of four different breast cancer cell lines [12], a finding which was replicated in both colon tumour and endothelial cells in the current study. This is in contrast to the report by Peng et al., who demonstrated the IC_50_ of bac I to be lower than that of bac II for multiple cancer cell lines [20]. One possible explanation for this discrepancy is the difference in the purity of bac I and II used. In our studies, bac I and II were commercially obtained and their reported purity was above 90%, while Peng et al. extracted both bac I and II from dried and powdered whole plant BM ‘in house’, and identified them by comparison of their spectral data with reported values in the literature [20]. The use of highly purified extracts prepared by consistent and reliable methods is paramount for ongoing studies using bac I and II.

The IC_50_ of both bac I and II varied significantly between the three cell lines examined, and so did the anti-tubulogenic effect of bac I and II combined between the two endothelial cell lines. HUVEC was the most sensitive, and even the lowest combination concentration of 5/2.5 µM completely inhibited their proliferation. HT-29 was the most resistant to cytotoxic effects; at the highest combination concentration of 10/5 µM, proliferation was only inhibited by 26.5% compared to the vehicle. Bac I/II 5/2.5 µM, which inhibited tube formation of 2H-11 by 63.4% compared to the vehicle, did not exhibit an anti-tubulogenic effect in HUVEC. Bac I and II combined only moderately reduced the migration of HT-29; bac I/II 10/5 µM inhibited its migration by 36.6% compared to the vehicle. Bac I and II combined appeared to be associated with a stronger anti-tubulogenic effect than antimigratory effect, probably because of the difference in the sensitivity to bac I and II between the three cell lines.

Multiple saponins have been described to induce apoptosis, and this is one of the proposed mechanisms underlying the cytotoxicity of BM [27,28,29]. Our group previously claimed treatment of MDA-MB-231, T-47D, MCF-7 and BT-474 breast cancer cells with bac I and II combined caused induction of cell death using an annexin V/PI staining assay [12]. Similar results have been shown on S-180 murine sarcoma cells following treatment with an ethanol extract of BM [17]. In the current work, we originally performed an annexin V/ PI staining assay on combined bac I and II treated HT-29, 2H-11 and HUVEC (Figure S1). This showed a significant proportion of annexin V-positive HUVEC to be PI-positive, raising the possibility that the finding may relate to loss of plasma membrane integrity rather than apoptosis. An additional literature review indicated non-apoptotic annexin V-positivity can result from binding of annexin V to still internalised phosphatidylserine (PS) when plasma membrane integrity is lost, even transiently, and from non-apoptotic externalisation of PS associated with tumour cells and endothelial cells in a tumour microenvironment [30,31,32,33]. We therefore proceeded with a caspase 3/7 apoptosis assay, which examines activated caspase 3/7, the universal executioner caspases in both the intrinsic and extrinsic apoptosis pathways.

The result of caspase 3/7 assay indicated no or minimal activation of caspase 3/7 in response to treatment with bac I and II, either alone or in combination, for HT-29 and 2H-11, at the concentrations and timeframe examined. Impaired proliferation observed for HT-29 and 2H-11 in the current work may be attributed to non-apoptotic cytotoxicity of bac I and II combined, or delayed induction of apoptosis, as the caspase 3/7 assay was terminated at 36 h, unlike the proliferation assay, which lasted for 72 h. One non-apoptotic cell death modality induced by combined bac I and II may be necroptosis, a programmed form of cell death which is inflammatory and caspase-independent, and some saponins have been reported to induce tumour cell death via necroptosis [34,35,36]. In contrast to HT-29 and 2H-11, caspase 3/7 was activated in HUVEC by all the combination concentrations tested. For those at 5/5 µM and above, the activation of caspase 3/7 was attained within 2 h of treatment application, being responsible for their anti-tubulogenic effect on this cell line. Impaired tube formation of 2H-11 was neither associated with apoptosis nor inhibition of proliferation at 24 h for bac I/II 5/2.5 and 10/2.5 µM, and the finding supports the non-apoptotic, non-proliferative anti-tubulogenic effect of combined bac I and II, at least for the low combination concentrations. A concentration-dependent increase in caspase 3 and 9 activity in response to powdered BM leaves has been reported in KB cells, a subline of HeLa cervical cancer cells [37].

While Ca^2+^ is vital to the physiological functioning of cells, Ca^2+^ overload or perturbation of intracellular Ca^2+^ compartmentalisation may cause cytotoxicity through induction of apoptosis, necrosis and autophagy [38]. An increase in cytosolic Ca^2+^ through saponin-induced pores has been proposed to activate Ca^2+^-dependent enzymes, leading to necrosis and apoptosis [39]. We observed an increase in cytosolic Ca^2+^ in response to treatment with bac I and II combined, in a dose dependent manner for all three cell lines. It has been proposed Ca^2+^ influx, mediated by plasma membrane localisation of mixed lineage kinase domain-like protein, is a crucial step in the aforementioned TNF-induced necroptosis [40]. Additionally, the cytosolic Ca^2+^ rise seen for HT-29 and 2H-11 was coupled with reduced wound closure and impaired tube formation, which involves cellular migration, respectively. The only exception was treatment of 2H-11 with bac I/II 5/2.5 µM, where the anti-tubulogenic effect of combined bac I and II was seen without associated cytosolic Ca^2+^ rise. It is possible that the interruption of intracellular Ca^2+^ dynamics, facilitated by combined bac I and II treatment, contributed to the impaired migration of HT-29 and tube formation of 2H-11 as Ca^2+^ is a major regulator of cellular motility [41]. Cytosolic Ca^2+^ flux in HT-29 did not always lead into inhibition of proliferation, unlike in 2H-11, although data were collected for a considerably longer period in the proliferation assay than the calcium flux assay. Future experiments should run for an extended period to assess whether the rise in cytosolic Ca^2+^ is reversed with time, and how it correlates with cell viability and proliferation.

Saponins are amphiphilic chemicals composed of one or more hydrophilic sugar parts and a lipophilic steroid or triterpenoid part. The interaction of saponins with the plasma membrane has been studied extensively, and they result in several sequelae: enhanced membrane permeability, membrane lysis, and alteration of plasma membrane dynamics and lateral organisation [39,42,43,44]. The various effects of saponins on the plasma membrane have been linked to their cytotoxicity through the induction of programmed cell death; specifically, apoptosis, necrosis and autophagy, which involve multiple mechanisms, including receptor activation, changes in ion channel permeability, and translocation of receptors or membrane proteins [39]. Serial monitoring for PI-stained cells in the current study revealed bac I and II combined induced the loss of plasma membrane integrity in 2H-11 and HUVEC, which was preceded by increased cytosolic Ca^2+^. In HUVEC, the rise of cytosolic Ca^2+^ occurred within 60 min, suggesting Ca^2+^ influx was mediated by the rapid activation of Ca^2+^ channels or transporters in the plasma membrane, or that of organelles such as the endoplasmic reticulum (ER). Alternatively, it might be mediated by the formation of pores too small for PI to pass through initially, or by the direct insult to ER. HT-29, on the other hand, did not display a loss of plasma membrane integrity when treated with bac I and II combined, at concentrations up to 10/5 µM, yet Ca^2+^ flux was still detected in this cell line. Ca^2+^ flux in HT-29 might be due purely to perturbation of intracellular Ca^2+^ or the activation of channels, pumps or transporters specific to Ca^2+^. John et al. reported treatment of LN-229 and U-87MG glioblastoma multiform cells with bacoside A, the major bioactive component of BM, induced more than a two-fold increase in intracellular free Ca^2+^ [45]. The finding was accompanied by an increase in phosphorylated calcium/calmodulin-dependent protein kinase IIA (CaMKIIA), which is implicated in intracellular Ca^2+^ homeostasis through the release of Ca^2+^ from ER via ryanodine and the inositol 1,4,5-trisphosphate receptors [46,47]. CaMKII is involved in ER stress-induced apoptosis, as well as mitochondrial-dependent apoptosis [48,49]. Whether Ca^2+^ rises occurred from extracellular influx, a release from intracellular organelles such as ER, or both, was not answered by our study.

Variation between cell lines was a recurrent theme throughout the findings in the current study. The effect of saponin on the membrane is influenced by several factors, including cholesterol content, which differs considerably between cell types and organelles, and the structure of saponins, specifically the type of saponin side chains and the nature of aglycone [19,39]. Bac I and II are proven modulators of aquaporin 1 (AQP1), a transmembrane protein with water and ion channel functions, and, as monotherapies, bac I and II have been shown to exhibit a significantly less antimigratory effect on AQP1 non-expressing colon cancer cell line SW480 compared to moderately AQP1-expressing HT-29 [14]. HUVEC, the most sensitive cell line to the antiproliferative and pro-apoptotic effect of bac I and II combined in the current study expresses a higher amount of AQP1 compared to HT-29, the most resistant cell line [50]. This supports the idea that the cytotoxicity of bac I and II combined is mediated through their action on the plasma membrane, and its composition influences their efficacy.

## 4. Materials and Methods

### 4.1. Reagents

The analytical standard chemical compounds bac I (Sigma-Aldrich, St. Louis, MO, USA; CAS No. 382148-47-2, ≥95% purity by HPLC) and bac II (Sigma-Aldrich; CAS No. 382146-66-9, ≥95% purity by HPLC) were dissolved in methanol (Sigma-Aldrich) at 10 mM and 1.5 mM, respectively, as stock solutions, and stored at −20 °C.

### 4.2. Cell Lines

HT-29 colon cancer cell line and 2H-11 mouse endothelial cell lines were purchased from the American Type Culture Collection (ATCC; Manassas, VA, USA), while HUVEC was purchased from Lonza (Basel, Switzerland). Cells were maintained in a complete medium, either Dulbecco’s modified Eagle’s medium (DMEM; Life Technologies, Carlsbad, CA, USA) containing 10% heat-inactivated fetal bovine serum (FBS) (Corning, NY, USA), Penicillin (100 U/mL), Streptomycin (100 µg/mL) (Life Technologies) and 2 mM L-alanyl-L-glutamine dipeptide (GlutaMAX Supplement; Life Technologies) for HT-29 and 2H-11 or EBM™-2 Endothelial Cell Growth Basel Medium, supplemented with EBM™-2 Endothelial SingleQuots™ kit (Lonza) for HUVEC. Cells were grown under standard culture conditions (37 °C with 5% CO_2_) and passaged no more than 5 times for HUVEC and no more than 20 times for HT-29 and 2H-11. All cell lines were mycoplasma-free, as determined using the MycoAlert™ Mycoplasma Detection Kit (Lonza) and/or a custom PCR-based assay, as described previously [11,51].

### 4.3. Cell Viability Assay to Determine IC_50_ and Drug Synergism

Cell viability was measured by a crystal violet assay, as described previously [13,50]. Briefly, cells were seeded at 1 × 10^3^ cells per well for HT-29 and 2H-11, and 3 × 10^3^ cells per well for HUVEC, on 96-well flat-bottomed plates and incubated overnight. Cells were treated for 24 h with a complete medium supplemented with vehicle (2% methanol) or various concentrations of bac I and/or II. Cells were fixed in formalin for 30 min, stained with 1% crystal violet in 2% ethanol for 10 min, eluted in 10% acetic acid for 1 h, and absorbance was measured at 595 nm. The number of cells seeded was optimised for cell size and doubling time of each cell line to avoid over confluency at the end of the assay.

The commonly used linear isobologram method was used to distinguish between synergistic, additive and antagonistic interactions between bac I and II [52,53,54]. First, IC_50_ with 95% confidence intervals was determined by nonlinear regression analysis for bac I and II alone. Next, the IC_50_ was determined for combined bac I and II. We elected to use a constant concentration of bac II at 5 μM for HT-29 and 2H-11, and 2.5 μM for HUVEC, whilst varying the concentration of bac I. A theoretical line was produced by plotting the IC_50_ and 95% confidence intervals for bac I and II alone on the y- and x-axis, respectively. The IC_50_ of bac I and II combined was plotted on the same graph, and its position relative to the theoretical line, above, on or below, indicated whether they were antagonistic, additive, or synergistic, respectively. CIx denoted whether the combination of bac I and II was antagonistic, additive or synergistic depending on whether CIx >, =, or <1, respectively. CIx was calculated as CIx = d1/Dx1 + d2/Dx2, where d1 and d2 were the concentrations of bac I and II, respectively, that, combined, gave an IC_50_; Dx1 was the IC_50_ of bac I alone, and Dx2 was the IC_50_ of bac II alone [52,53,55].

RSM was applied to confirm the synergism between bac I and II using the results of HUVEC cell viability. The central composite design technique was employed with 3 levels; low, mid, and high values, which were concentrations of bac I and II, corresponded to −1, 0 and +1 for input. The concentration ranged from 0–10 µM for bac I and 0–5 µM for bac II. Table S1 summarises the values selected for low, mid, and high bounds of concentrations for bac I and II.

### 4.4. Cell Proliferation Assay

Cellular proliferation was measured to assess the antiproliferative effect of bac I and II, either alone or in combination, on HT-29, 2H-11 and HUVEC using a crystal violet assay, as described previously [11,56]. Briefly, cells were seeded in complete medium at 1 × 10^3^ cells per well for HT-29 and 2H-11, and 3 × 10^3^ cells per well for HUVEC on 96-well flat-bottomed plates, respectively. After overnight incubation, treatment with vehicle or bac I and/or II was commenced, and crystal violet absorbance measured at 595 nM on days 0, 1 and 3 of treatment. The number of cells seeded was optimised for cell size and doubling time of each cell line to avoid over confluency at the end of the assay.

### 4.5. Circular Scratch Wound Migration Assay

Cellular migration was measured to assess antimigratory effect of bac I and II either alone or in combination on HT-29 by circular scratch wound migration assay as described previously [13,57]. Briefly, HT-29 cells were seeded in a complete medium at 1 × 10^5^ cells per well on 96-well flat-bottomed plates and incubated under the standard culture condition until 80% confluent. The medium was replaced with a serum-reduced medium and cells were incubated overnight. A circular wound was made on the cellular monolayer using a 10 µL pipette tip. The serum-reduced medium was changed to a complete medium, supplemented either with vehicle or bac I and/or II, and, to inhibit cell proliferation, 1 μg/mL mitomycin C (Sigma-Aldrich, St. Louis, MO, USA). Images were captured at time 0 and after 48 h on an Eclipse TE2000-U light microscope (Nikon, Tokyo, Japan) at 40× magnification. NIS-Elements BR software (Nikon) was used to measure the area of the wound relative to the area at time 0.

### 4.6. Tube Formation Assay

A tube formation assay was performed to assess the anti-angiogenic effect of bac I and II, either alone or in combination, on 2H-11 and HUVEC, as previously described [13,58]. Briefly, 96-well angiogenesis μ-plates (Ibidi, Martinsried, Germany) were prepared by coating wells with Matrigel (Corning), according to the manufacturer’s instructions. In the respective complete medium, 2H-11 and HUVEC were resuspended, supplemented with either vehicle or bac I and/or II, and were plated at 1.5 × 10^4^ cells per well. After 2-h incubation, the number of loops formed was counted.

### 4.7. Caspase-3/7 Apoptosis Assay

Activation of caspase 3 and 7 was assessed to determine the induction of apoptosis in HT-29, 2H-11 and HUVEC by bac I and II, either alone or in combination, using CellEvent™ Caspase-3/7 Green Detection Reagent (Thermo Fisher Scientific, Waltham, MA, USA), according to the manufacturer’s instruction. Cells were seeded at 3.3 × 10^3^ cells for HT-29 and 1.5 × 10^3^ cells for 2H-11 and HUVEC per well on 96-well flat-bottomed plates. The following day, cells were treated with vehicle or bac I and/or bac II containing 1:1000 dilution of Caspase-3/7 Green Detection Reagent. Staurosporine 0.25 µM (Sigma-Aldrich) was used as the positive control. The number of caspase 3/7-positive (green) cells was determined using an IncuCyte S3 Live-Cell Analysis System (Sartorius, Goettingen, Germany), acquiring 4 images per well every 2 h for 36 h.

### 4.8. Calcium Flux Assay

Calcium flux was measured using a Fluo-8 assay (AAT Bioquest, Sunnyvale, CA, USA) to assess the effect of bac I and II, either alone or in combination, on cytosolic Ca^2+^ in HT-29, 2H-11, and HUVEC, as per the manufacturer’s instruction. Briefly, cells were seeded in the respective complete medium at 2 × 10^4^ cells per well on 96-well flat-bottomed plates and incubated overnight. Cells were labelled with a dye working solution consisting of Hank’s and HEPES Buffer (HHBS), supplemented with 0.08 % pluoronic^®^ F-127 (AAT Bioquest), 4 mM probenecid (AAT Bioquest) and 4 µM Fluo-8. Pluoronic^®^ F-127 and probenecid were used to improve aqueous solubility of AM esters attached to Fluo-8 and to reduce the leakage of the de-esterified indicators, respectively. The plates were incubated for 30 min at 37 °C, followed by a 30-min incubation at room temperature. Media was replaced with HHBS containing 1 mM probenecid and vehicle or bac I and/or II, and fluorescence was measured at time 0, 10 and 30 min, 1, 1.5, 2, 3 and 6 h on a FLUOstar OPTIMA microplate reader (BMG Labtech, Ortenberg, Germany).

### 4.9. Propidium Iodide Assay to Assess Plasma Membrane Integrity

Plasma membrane integrity of HT-29, 2H-11 and HUVEC, in response to treatment with bac I and II, either alone or in combination, was assessed using PI, which is unable to permeate an intact cell membrane. Cells were seeded at 3.3 × 10^3^ cells per well on 96-well flat-bottomed plates. The following day, cells were treated with vehicle or bac I and/or bac II in the respective complete medium containing 2.5 μg/mL PI (Sigma-Aldrich). The number of PI-positive cells was determined using an IncuCyte S3 Live-Cell Analysis System, acquiring 4 images per well every 2 or 3 h for 24 h.

### 4.10. Statistical Analysis

Data were analysed and IC_50_ determined using GraphPad Prism version 8.4.3 (GraphPad Software, La Jolla, CA, USA). For proliferation, caspase 3/7, calcium flux and PI assays, mixed-effects analysis, with repeated measures and Dunnett’s multiple comparisons, was employed with respect to vehicle. For the remaining experiments, a one-way analysis of variance (ANOVA) with Dunnett’s multiple comparisons was performed with respect to vehicle. Statistical significance was set at *p* < 0.05.

## 5. Conclusions

The current study demonstrated that extracts of BM, bac I and bac II synergistically inhibit the viability of HT-29 colon cancer, and 2H-11 and HUVEC endothelial cells. Bac I and II combined additionally exhibited antiproliferative, antimigratory and anti-tubulogenic properties, with more overall potency in endothelial cells. The findings support further preclinical testing of combined bac I and II using animal models, and the potential repurposing of these herbal extracts as cancer therapeutics. Mechanisms underlying their cytotoxicity differed between cell lines. Induction of apoptosis was only apparent in HUVEC, while a surge in cytosolic Ca^2+^ was demonstrated in all the cell lines, suggesting it is involved in both apoptotic and non-apoptotic cell death induced by bac I and II combined. How Ca^2+^ enters cytosolic space is yet to be elucidated; however, disturbance in plasma membrane integrity is thought to play a role at least in endothelial cells. Further research is required to better understand the anticancer mechanisms, and the in vivo efficacy, of bac I and II.

## Figures and Tables

**Table 1:** The IC_50_ of bac I and II alone and combined for HT-29, 2H-11 and HUVEC.

Cells	Bac I (95% CI) (µM)	Bac II (95% CI) (µM)	Bac I (95% CI)/II Combined (µM)
HT-29	97.9 (82.7–115.9)	20.6 (19.0–22.3)	6.8 (6.4–7.3)/5
2H-11	105.7 (99.0–112.9)	12.4 (12.1–12.7)	9.5 (8.4–10.8)/5
HUVEC	29.2 (23.7–35.9)	4.5 (4.1–5.0)	4.6 (2.5–8.5)/2.5

## Data Availability

All relevant data are provided within the manuscript and the supplementary materials.

## Supplementary Materials

The following are available online at https://www.mdpi.com/article/10.3390/ph14050436/s1: Table S1: Low, mid and high values used as bounds of concentrations for bacopaside I and II in the RSM analysis; Table S2: The designed matrix used in the RSM analysis; Figure S1: Bacopaside I and II combined induced annexin V positivity in HT-29, 2H-11 and HUVEC.
