# Supplementary material for: In Vitro Synergistic Inhibition of HT-29 Proliferation and 2H-11 and HUVEC Tubulogenesis by Bacopaside I and II Is Associated with Ca2+ Flux and Loss of Plasma Membrane Integrity
Source: Pharmaceuticals (Basel). 2021 May 6;14(5):436. doi: 10.3390/ph14050436 (PMC8148107; doi:10.3390/ph14050436)
Supplement: Supplementary file 1 [file pharmaceuticals-14-00436-s001.zip › pharmaceuticals-1163207-SI.pdf]

pharmaceuticals

Table S1. Low, mid and high values used as bounds of concentrations for bacopaside I and II in the RSM analysis.

Parameter	Index	Concentration (μM)		
		Low value (-1)	Mid value (0)	High value (+1)
Bac I	A	0	5	10
Bac II	B	0	2.5	5

Table S2. The designed matrix used in the RSM analysis.

Run	Index value		Concentration (μM)		Response (%)
	A (bac I)	B (bac II)	Bac I	Bac II	
1	-1	0	0	2.5	80.75
2	0	0	5	2.5	60.67
3	0	0	5	2.5	42.68
4	0	0	5	2.5	60.67
5	1	0	10	2.5	33.89
6	1	-1	10	0	84.94
7	0	0	5	2.5	47.28
8	0	0	5	2.5	46.86
9	0	1	5	5	34.31
10	-1	-1	0	0	100
11	0	-1	5	0	53.97
12	-1	1	0	5	79.92
13	1	1	10	5	10.04

The response was expressed as cell viability of HUVEC treated with bac I and II either alone or in combination, relative to vehicle in percentage. To optimize the combination of concentrations, the RSM model has reduced the total experiments to 13 iterations, with cell viability being the "main measurable target parameter".

Figure S1. Bacopaside I and II combined induced annexin V positivity in HT-29, 2H-11 and HUVEC.

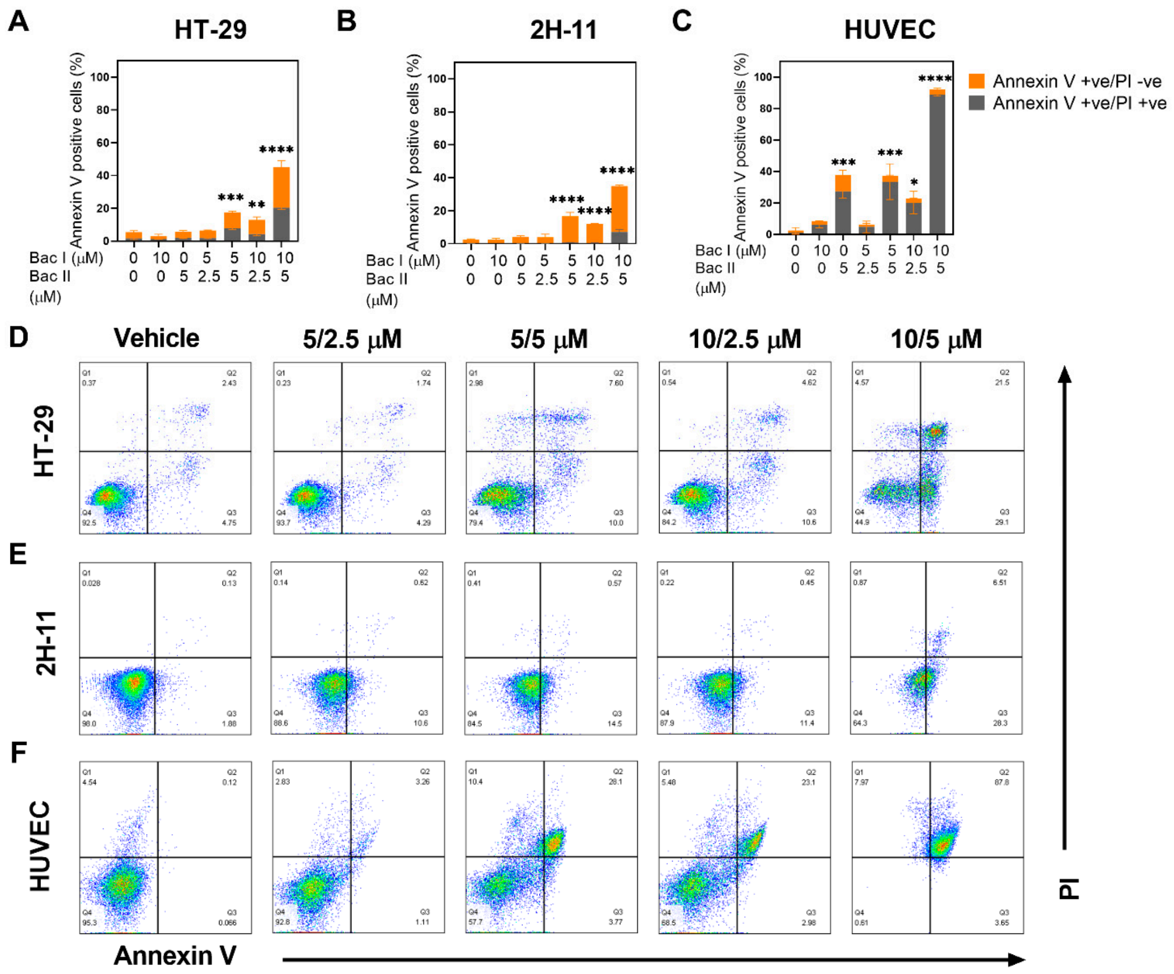

HT-29 (A & D), 2H-11 (B & E) and HUVEC (C & F) were treated with bac I and II either alone or in combination, and annexin V/PI staining performed. In bar graphs (A, B & C), data represents the mean percentages of annexin V-positive/PI-negative (orange bars) and annexin V-positive/PI-positive (grey bars) cells for triplicates. The scatter plots (D, E & F) show population gates of annexin V-negative/PI cells (left lower quadrant), annexin V-positive/PI-negative cells (right lower quadrant), annexin V-positive/PI-negative cells (right upper quadrant), and annexin V-negative/PI-positive cells (left upper quadrant). Error bars represent SD and significant difference as compared to vehicle is indicated by asterisks (** $p < 0.01$; *** $p < 0.001$; **** $p < 0.0001$).
